# On the Identification of Orthotropic Elastic Stiffness Using 3D Guided Wavefield Data

**DOI:** 10.3390/s22145314

**Published:** 2022-07-15

**Authors:** Adil Han Orta, Mathias Kersemans, Koen Van Den Abeele

**Affiliations:** 1 Wave Propagation and Signal Processing (WPSP), Department of Physics, KU Leuven—Campus Kulak, 8500 Kortrijk, Belgium; koen.vandenabeele@kuleuven.be; 2 Mechanics of Materials and Structures (MMS), Department of Materials, Textiles and Chemical Engineering, Ghent University, Technologiepark 46, 9052 Ghent, Belgium; mathias.kersemans@ugent.be

**Keywords:** material characterization, non-destructive testing, particle swarm optimization, orthotropy, Lamb waves, composites, woods

## Abstract

Scanning laser Doppler vibrometry is a widely adopted method to measure the full-field out-of-plane vibrational response of materials in view of detecting defects or estimating stiffness parameters. Recent technological developments have led to performant 3D scanning laser Doppler vibrometers, which give access to both out-of-plane and in-plane vibrational velocity components. In the present study, the effect of using (i) the in-plane component; (ii) the out-of-plane component; and (iii) both the in-plane and out-of-plane components of the recorded vibration velocity on the inverse determination of the stiffness parameters is studied. Input data were gathered from a series of numerical simulations using a finite element model (COMSOL), as well as from broadband experimental measurements by means of a 3D infrared scanning laser Doppler vibrometer. Various materials were studied, including carbon epoxy composite and wood materials. The full-field vibrational velocity response is converted to the frequency-wavenumber domain by means of Fourier transform, from which complex wavenumbers are extracted using the matrix pencil decomposition method. To infer the orthotropic elastic stiffness tensor, an inversion procedure is developed by coupling the semi-analytical finite element (SAFE) as a forward method to the particle swarm optimizer. It is shown that accounting for the in-plane velocity component leads to a more accurate and robust determination of the orthotropic elastic stiffness parameters.

## 1. Introduction

The identification of elastic stiffness parameters is fundamental for structural design and health monitoring. Design recommended stiffness parameters might change over time due to cyclic thermal or mechanical exposure, or as the result of an abrupt impact event. Therefore, the implementation of fast and accurate characterization methods is essential in non-destructive evaluation (NDE) and structural health monitoring (SHM) [1]. In view of this, there are two types of non-destructive techniques that are commonly used to identify stiffness parameters, namely vibrational and wave propagation methods [2].

In vibration-based methods, the specimen is excited via a shaker or a transducer, and the frequency response at different points on the surface is measured. The measured frequency response is then used to extract modal parameters, such as natural frequencies, modal damping and mode shapes, which are analyzed to identify the stiffness parameters [3,4]. However, as the extraction of modal parameters in the high frequency range and the realization of appropriate boundary conditions become quite complex, small errors in the acquisition of the raw data and in the geometry and/or set-up might lead to high errors in the inverted stiffness parameters.

In wave propagation methods, a pair of transducers consisting of an emitting source and a receiving sensor is used to send and record an ultrasonic wave. The propagation behavior of the generated wave obviously depends on the amplitude and frequency content of the excitation signal, as well as on the geometrical, structural and elastic properties of the medium. In recent decades, a range of forward models were developed in the literature to predict wave propagation in various media and geometries, and to inversely estimate the genuine stiffness parameters. Among wave propagation-based characterization methods, the use of bulk waves in through-transmission [5,6,7] or Lamb waves along the plate [8,9,10,11,12] is well recognized. Both classes of methods essentially use the phase velocity or group velocity measurements of a propagating ultrasonic wave to evaluate the stiffness tensor of the material [13].

Although predictive bulk wave models are computationally efficient, material characterization with bulk waves has several limitations. Firstly, the bulk wave approximation is only valid in a specific frequency range. The ultrasound wavelength (λ) has to be far smaller than the plate thickness (*d*) because of the infinite plate assumption, placing a lower boundary on the frequency range. Additionally, damping effects and fundamental homogenization conditions put an upper boundary for the “frequency × thickness” (*fd*) regime. Secondly, the effects of plate boundaries are neglected due to the inherent infinite thickness assumption which might lead to discontinuous phase shifts in transmitted signals. Even though bulk waves are independent of the frequency and plate thickness, abrupt layer transitions at interfaces lead to phase changes that need to be accounted for in calculating the time-of-flight as well as in the inversion of the stiffness constants [7]. Third and finally, a priori knowledge of the material symmetry axes is necessary for the bulk wave-based characterization methods. In the case of misalignment, the mismatch between numerical and experimental propagation directions might result in large errors in the inverted stiffness parameters [7,14]. As a valuable alternative, Lamb waves can be used to overcome the drawbacks of a bulk wave-based characterization method. However, the forward model to calculate the Lamb waves in multilayered composite structures is more sophisticated and inversions based on such models obviously require more computational power.

With respect to the acquisition of vibrational and wave propagation signals, laser Doppler vibrometry (LDV) is a widely adopted approach to measure the local vibrational response of materials. In the simplest LDV device set-up, the recorded data contain information about the out-of-plane velocity response at a single point. To increase the backscattered laser signal and as such to improve the signal-to-noise ratio, surface preparation (e.g., retro-reflective tape or small glass beads) is often required. If the full-field vibrational response is of interest, the structure needs to be successively measured at a multitude of points by performing a mechanically operated scan, e.g., by means of a robotic arm. More advanced measurement systems use a scanning laser Doppler vibrometer (SLDV) which enables access to full-field velocity data in a much shorter time, which Are crucial for SHM and NDE [10,11]. Furthermore, the combined use of three measurement lasers, each measuring the 3D displacement at a single material spot from different orientation, allows to extract both the out-of-plane velocity components and the two in-plane contributions simultaneously. Finally, the adoption of infrared lasers significantly improves the backscattered signal of obliquely incident laser beams, resulting in high-quality measurements even without surface preparation. Hence, the use of such a 3D infrared SLDV system is highly recommended to quickly and reliably access the full-field in-plane and out-of-plane vibrational response of materials in a wide frequency range [15].

In the present study, the weight of the information contained in the in-plane surface motion on the inversion of the material’s stiffness parameters via Lamb wave-based characterization is investigated. The inversion procedure is repeatedly conducted by adding or ignoring the in-plane components of the vibration velocity data, and the error levels as well as the median absolute deviations are evaluated. To verify the accuracy of the inversion, a series of numerical simulations using a finite element model (COMSOL) for different materials with selected elasticity values are conducted. The received signal data from these simulations are subsequently converted into the frequency-wavenumber domain by means of the matrix pencil decomposition method. As a forward model to be used in the inversion process, the semi-analytical finite element (SAFE) method is used because of its accuracy, robustness and computational efficiency. The SAFE method is then combined with the particle swarm optimization procedure for the inversion. The inversion procedure is repeated 20 times to avoid local minima and to obtain statistics on the results. Following the numerical validation, the inversion procedure is experimentally demonstrated on a carbon epoxy (C/E) and G/F woven plate by conducting the same analysis procedure on data acquired using a 3D infrared scanning laser Doppler vibrometer. The obtained results show that in-plane motion components are essential to obtain a more accurate and robust reconstruction of the elastic parameters.

The paper is structured as follows. In Section 2, the mathematical model of the forward model (SAFE) and the inversion routine based on the particle swarm optimizer are briefly recapitulated. In Section 3, the measurement protocols for numerical simulations using COMSOL and for the experimental data are outlined. In addition, details of the matrix pencil decomposition method are shared. In Section 4, inversion results for the numerical studies as well as for the experiments are presented for different materials. Moreover, the effect of the noise on the inverted stiffness parameters and on the median absolute deviations are investigated. Finally, some conclusions of this study are summarized in Section 5.

## 2. Optimization Procedure

### 2.1. Forward Model SAFE

The semi-analytical finite element method (SAFE) is an approximate method to model the propagation of elastic waves in a medium. This method uses the infinite plate assumption to ignore reflections coming from boundaries and employs far-field solutions to ignore transducer effects. In the SAFE method, the material is firstly discretized in the through thickness direction to formulate the stiffness and mass matrices [16,17]. Then, energy equilibrium is used, resulting in a generalized eigenvalue problem. This approach overcomes several drawbacks of conventional 3D elasticity-based methods, such as the root searching problem and the occurrence of numerical instabilities [16].

For the calculation of wavenumbers with the SAFE method, it is known that 1D iso-parametric elements provide good results [16]. To do this, displacements per element (indicated by superscript *e*) $u^{\left(e\right)}\left(x,y,z,t\right)$ are expressed in terms of shape functions, $N_{j}\left(y,z\right)$, and unknown nodal displacement components, ($U_{xj}$, $U_{yj}$, $U_{zj}$), as shown in Equation (1).

(1) $$\begin{array}{c} u^{\left(e\right)}\left(x,y,z,t\right)=\left[\begin{array}{c} \sum_{j=1}^{n}N_{j}\left(y,z\right)U_{xj}\\ \sum_{j=1}^{n}N_{j}\left(y,z\right)U_{yj}\\ \sum_{j=1}^{n}N_{j}\left(y,z\right)U_{zj} \end{array}\right]e^{i(kx-\omega t)}=N\left(y,z\right)q^{\left(e\right)}e^{i(kx-\omega t)} \end{array}$$

Here, *k* is the wavenumber, ω is the angular frequency, and *n* is the number of nodes per element. By using Equation (1), the strain components ε can be expressed as a function of the nodal displacements:

(2) $$\varepsilon =\left[L_{x}\frac{\partial }{\partial x}+L_{y}\frac{\partial }{\partial y}+L_{z}\frac{\partial }{\partial z}\right]N\left(y,z\right)q^{\left(e\right)}e^{i(kx-\omega t)}=\left(B_{1}+ikB_{2}\right)q^{\left(e\right)}e^{i(kx-\omega t)}$$

where $q^{\left(e\right)}$ is the unknown nodal displacement for each element, $B_{1}=L_{y}N_{,y}+L_{z}N_{,z}$, $B_{2}=L_{x}N$. $N_{,y}$ and $N_{,z}$ variables are the derivatives of the shape function matrix with respect to the *y* and *z* directions, respectively. The matrix *L* represents the strain parameters in matrix form as expressed in Bartoli et al. [16]. Using this formalism, the stiffness and mass matrices for each element can be calculated as follows:

(3) $$\begin{array}{ccccc} k_{1}^{\left(e\right)} & =\int_{\Omega_{e}}^{}\left[B_{1}^{T}{\tilde{C}}_{e}B_{1}\right]d\Omega_{e}, & k_{2}^{\left(e\right)} & =\int_{\Omega_{e}}^{}\left[B_{1}^{T}{\tilde{C}}_{e}B_{2}-B_{2}^{T}{\tilde{C}}_{e}B_{1}\right]d\Omega_{e} & \\ k_{3}^{\left(e\right)} & =\int_{\Omega_{e}}^{}\left[B_{2}^{T}{\tilde{C}}_{e}B_{2}\right]d\Omega_{e}, & m^{\left(e\right)} & =\int_{\Omega_{e}}^{}\left[N^{T}\rho_{e}N\right]d\Omega_{e}, \end{array}$$

The mass ($m^{\left(e\right)}$) and stiffness ($k_{1}^{\left(e\right)},k_{2}^{\left(e\right)}$ and $k_{3}^{\left(e\right)}$) matrices for each element need to be combined in corresponding global matrices to compute the dynamic response of the entire material’s through thickness behavior. The global matrices can be evaluated as follows:

(4) $$\begin{array}{cccccccc} K_{1} & =\overset{n_{el}}{\underset{e=1}{⋃}}k_{1}^{\left(e\right)}, & K_{2} & =\overset{n_{el}}{\underset{e=1}{⋃}}k_{2}^{\left(e\right)}, & K_{3} & =\overset{n_{el}}{\underset{e=1}{⋃}}k_{3}^{\left(e\right)}, & M & =\overset{n_{el}}{\underset{e=1}{⋃}}m^{\left(e\right)} \end{array}$$

where $n_{el}$ is the total number of cross-sectional elements. By considering these global matrices and imposing energy equilibrium, the homogeneous wave equation can be written as:

(5) $${\left[K_{1}+ikK_{2}+k^{2}K_{3}-\omega^{2}M\right]}_{M}U=0$$

The solution of this generalized eigenvalue problem (Equation (5)) provides the wavenumber values from which the dispersion curves can be obtained. The wavenumber values also yield the phase velocities which directly link to the elastic stiffness tensor.

### 2.2. Inversion Optimizer PSO

The selection of a suitable optimization model depends on several factors such as the dimension, bounds and topology of the parameter space [2]. For the inversion of the stiffness tensor of orthotropic materials, having nine independent elastic parameters, gradient-based optimization methods turn out to be inconvenient because of the notorious likelihood of converging to a local minimum. Alternatively, a heuristic algorithm generally has a much better ability to attain a stable inversion of the stiffness properties. Therefore, in the literature, there has been a considerable interest in using and evaluating a quite broad spectrum of optimization algorithms such as the genetic algorithm [10,11], particle swarm optimization [7,18], simulated annealing optimization [19], and surrogate optimization [20]. In a previous work, the accuracy and computational efficiency of different optimization algorithms have been compared for applications related to material characterization, and the results revealed that the stiffness tensor values closest to the targeted values were obtained by particle swarm and simulated annealing. However, in terms of computational speed, the particle swarm algorithm was superior compared to simulated annealing [20]. Therefore, in the present study, the particle swarm optimization was adopted due to its computational efficiency and high performance.

Particle swarm optimization (PSO) is a heuristic optimization method which is inspired by the study of bird flock preying behavior [21]. Because of the heuristic approach of PSO, the objective function does not need to be differentiable. Instead, the algorithm assumes that each individual particle (representing a combination of outcome parameters) in the search space has a position, speed, and fitness value calculated by the objective function. The algorithm is first initialized based on a random population of particles. Then, the best position of the swarm and of each particle are used as a guide to move around in the search space until the global minimum is found. For the implementation of PSO, the MATLAB Global Optimization Toolbox™ is used. In addition, the swarm optimization can be combined with the constrained nonlinear multivariable function ‘fmincon’ in order to create a hybrid optimization in view of improving the convergence and accuracy for finding the global minimum. The selected optimization variables and search space bounds for the present study are adopted as follows:

- Lower Bound: −40% of literature values;
- Upper Bound: +60% of literature values;
- Maximum Iterations—50;
- Swarm Size—50;
- Maximum Stall Iterations—20;
- Interior-point Algorithm (fmincon);
- Maximum Iterations (fmincon)—1000.

### 2.3. Inversion Procedure

**Figure 1:**
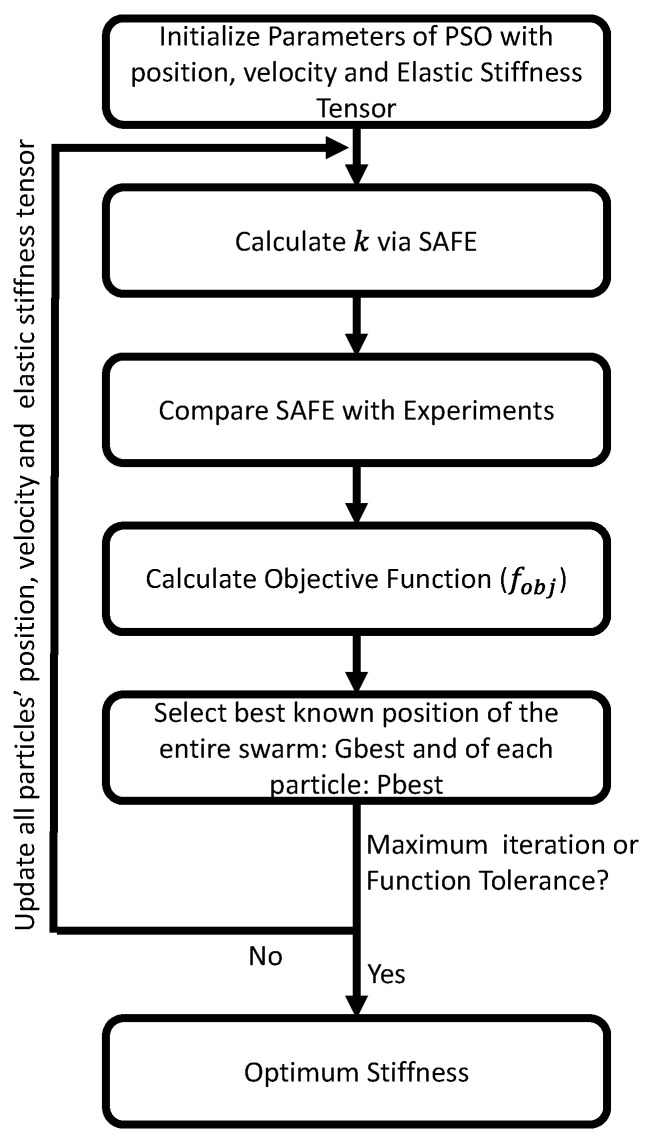
Flowchart of the inversion procedure to determine the elastic stiffness parameters. This entire procedure is repeated 20 times in each case study to obtain median values and median absolute deviations on the output parameters.

Following the selection of SAFE as a forward model and PSO as an inverse model, the actual inversion starts with a randomly selected swarm of stiffness values and iteratively optimizes the swarm characteristics. To do this, an objective function is selected as the sum of the absolute percentage error between the experimentally determined wavenumbers (based on the recorded data) and the numerically calculated wavenumbers (based on the SAFE method), and is expressed as follows:

(6) $$f_{obj}=\sum_{i=1}^{F}\sum_{j=1}^{K}\sum_{l=1}^{L}|\frac{k_{exp}^{i,j,l}-k_{num}^{i,j,l}}{k_{exp}^{i,j,l}}|$$

where *F* stands for the total number of frequencies used, *K* stands for the total number of wave modes, and *L* stands for the total number of propagation directions (ϕ). To calculate the objective function, each experimental wavenumber obtained from MPDM is compared to the closest theoretically predicted wavenumber value. The inverse model iteratively tries to minimize the error between the measured and simulated wavenumbers by changing the stiffness parameters. It is also possible to include weights for each stiffness parameter to increase the probability of convergence in consideration of the fact that certain stiffness parameters are more or less sensitive for certain wave modes in certain propagation directions [10]. However, as the selection of these weights requires a sensitivity study as a pre-processing step, and the weights need to be changed accordingly for different materials, weighting is not used in this study. The flowchart summarizing the implemented inversion procedure can be found in Figure 1. The entire procedure is repeated 20 times and the median values are reported as optimized stiffness parameters. To evaluate the robustness of the inversion method, the median absolute deviation for each stiffness parameter is reported.

## 3. Data Generation and Conditioning

In order to validate the accuracy and robustness of the inversion procedure, several synthetic datasets of wave propagation in materials with an a priori known stiffness tensor are considered. In addition to these artificial datasets created by finite element, several wave propagation experiments on various materials with a non a priori known elasticity were conducted (only estimates are available). The simulated or experimentally measured values are subsequently used in the same exact manner as an input for matrix pencil decomposition method (MPDM) to extract the appropriate wavenumbers. The following sections provide some details about these actions.

### 3.1. Simulation Data—COMSOL

**Figure 2:**
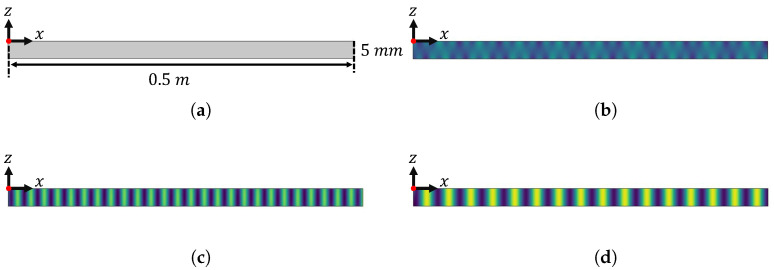
Model parameters and results of the numerical simulation: (**a**) The geometry and size of the plate in the two-dimensional finite element model, together with the source position; (**b**) The displacement field in *x* direction (**b**), in *y* direction (**c**), and in *z* direction (**d**) for an aluminum plate with 5 mm thickness at 100 kHz for an in-plane propagation angle ϕ = 0.

While it would have been possible to use SAFE as the forward model to create an artificial dataset for the inversion, an independent and comprehensive finite element method should be used as an alternative, within a fully controlled environment, to create a synthetic dataset where the geometric properties as well as the stiffness parameters can be defined by the user. As such, the “inverse crime” problem is avoided [22], and the targeted stiffness values to compare the outcome of the inversion to are exactly known. However, creating a 3D finite element model to calculate the wave propagation behavior of Lamb waves in plate-like structures over an extended frequency range is quite a challenging task. First of all, as suggested in the literature, the number of elements should be between 10 and 20 elements per wavelength [23]. Secondly, the size of the plate also needs to be large enough to avoid near source transient effects and to observe stabilized wave dynamics. This typically results in millions of degrees of freedom that need to be solved, which is quite challenging in terms of computational power and speed. Instead of fully 3D simulations, it is possible to create and analyze the outcome of 2D models, but these models can only be used for wave propagation along the principal directions in an orthotropic medium due to the dominant shearing effect in certain propagation directions. To overcome these problems, a 2D semi-analytical finite element model [24] was implemented in the Partial Differential Equation Solver of COMSOL Multiphysics. The geometry and dimensions of the model are displayed in Figure 2a, and are fixed for all simulations. The plate is excited from the top left corner of the plate, indicated with a red dot on the figure. The top and bottom surface of the plate are assumed to be stress-free boundaries ($\sigma_{13}=\sigma_{23}=\sigma_{33}=0$), and the boundary conditions in the rest of the plate are assumed to be motion free. A quadrilateral mesh with an element size of 1 mm is used with Lagrangian shape functions and a cubic element order. As such, using five elements in the through thickness direction is sufficient to obtain accurate results for the selected frequency regime. In total, the model employs a mesh of 2500 elements with more than 140,000 degrees of freedom. The simulations were performed using the frequency-domain finite element method because of its computational efficiency. It only takes a couple of minutes to solve this forward model on a workstation with Intel^®^Core™i7-8700 CPU @ 3.20 GHz and 32 GB ram. Nonetheless, if these finite element simulations were to be used for a material characterization inversion process, it might require days to identify the orthotropic material properties in a single case study. Viscoelastic effects can also be modeled with this method, but these are not considered in this study.

Figure 2b–d displays the computed wave field displacement in a 5 mm-thick aluminum plate at a frequency of 100 kHz. For an isotropic medium, the wave field is independent of the in-plane propagation direction. In the case of simulating orthotropic materials, the simulations are repeated for different propagation directions (ϕ) between 0 and 90 degrees with a 15-degree step. In each case, the in-plane and out-of-plane velocity signals at the top surface of the plate are recorded.

### 3.2. Experimental Data—3D IR SLDV

**Figure 3:**
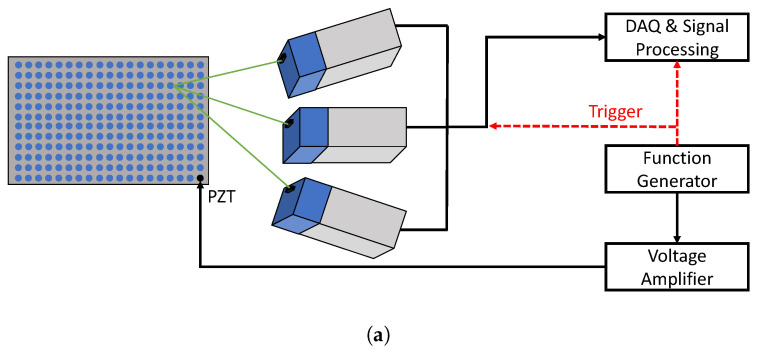

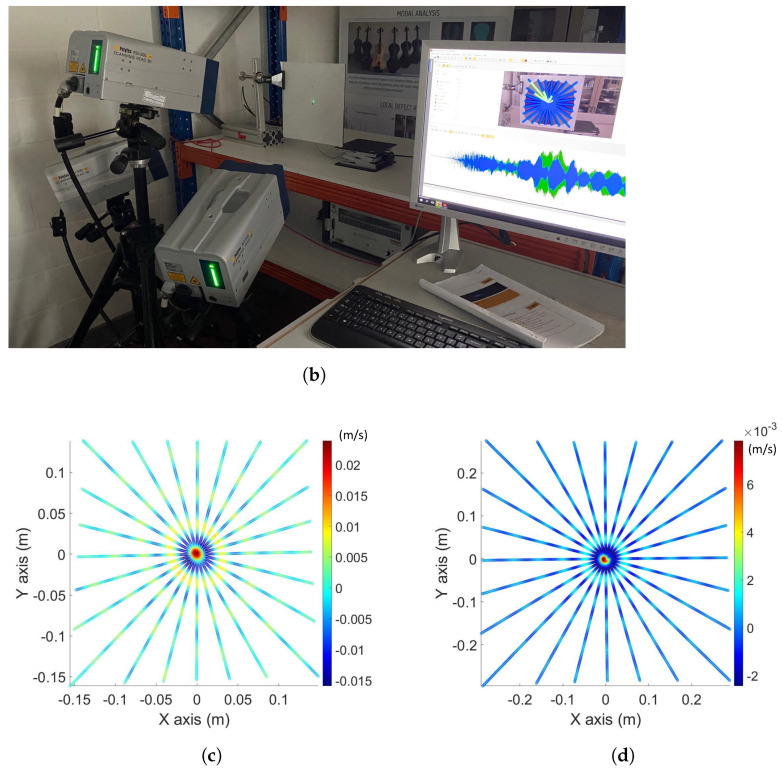
Schematic of the surface measurements and recording of wave propagation using a 3D infrared SLDV: (**a**) schematic of the experimental procedure; (**b**) experimental setup; (**c**) the recorded wave propagation on a C/E layered plate at *t* = 1.2 ms along a discrete set of angles; and (**d**) the recorded wave propagation on a G/F woven plate at *t* = 1.2 ms along a discrete set of angles.

To experimentally validate the performance of the proposed inversion method, actual wave propagation measurements are performed on a 330 × 330 × 2 mm${}^{3}$ carbon epoxy (C/E) with stacking sequence [45/0/–45/90]${}_{s}$ and a 600 × 600 × 6 mm${}^{3}$ G/F woven plate with a stacking sequence [#(45/–45)/#(0/90)]${}_{3s}$. A piezoelectric patch (type EPZ-20MS64W) at the center of each plate is used as an actuator. A Falco systems WMA-300 voltage amplifier was employed to supply a 100 $V_{pp}$ voltage to the actuator. For both plates, a broadband sine sweep signal between 5 kHz and 300 kHz is applied. The full-field (3D) vibrational response at the surface of these plates is recorded using a 3D infrared SLDV (Polytec PSV-500-3D-Xtra) (see Figure 3a,b ) with a sampling frequency of 625 kS/s. The C/E layered plate and the G/F woven plate are scanned by the SLDV with grid step $D_{x}=D_{y}=1$ mm and $D_{x}=D_{y}=1.25$ mm, respectively. Figure 3c,d visualize snapshots of the recorded vibrational response of both plates in a star-like layout corresponding to a discrete set of angles (Δϕ = 15 degrees). In total, for C/E and G/F woven plates, 3801 and 5997 points are measured, respectively. The recorded dataset is subsequently converted into the frequency-wavenumber domain by means of a 3D fast Fourier transform.

### 3.3. Matrix Pencil Decomposition Method (MPDM)

As the inversion procedure operates on wavenumber data, additional post-processing steps are required on the synthetic and actual experimental full-field velocity datasets to estimate the wavenumbers. The most commonly used methods described in the literature are the Fourier transform [25], the time–frequency analysis [26], the zero-crossing technique [27], the matrix pencil decomposition method [28], the sparse wavenumber analysis [29], the spatial Laplace transform [30], the inhomogeneous wave correlation [31], and the estimation of signal parameters via rotation invariant technique (ESPRIT) [18].

**Figure 4:**
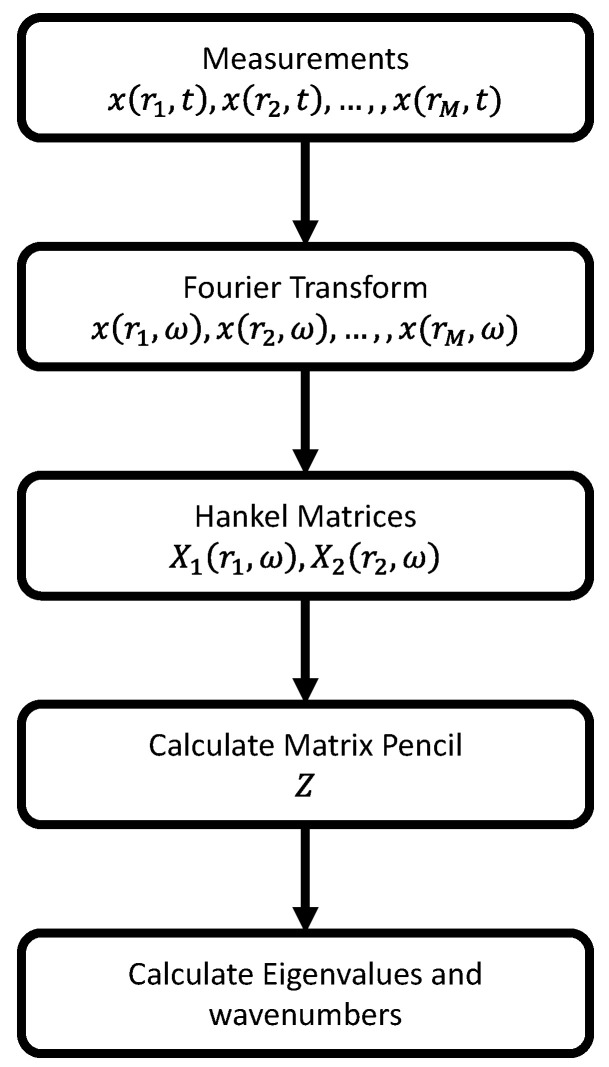
Flowchart of the matrix pencil procedure applied to estimate the pertinent wavenumbers.

Among the various wavenumber extraction methods, the matrix pencil decomposition method (MPDM), the estimation of signal parameters via rotation invariant technique (ESPRIT), and the inhomogeneous wave correlation (IWC) method stand out due to their advantages especially for complex wavenumber extraction. These methods were already being intensely used to identify the elastic and damping properties of materials in several studies [18,28,32]. MPDM and ESPRIT used singular value decomposition and the eigen-decomposition of the multi-channel covariance matrix, respectively. The IWC method minimizes the difference between the measured and simulated wave propagations by simply optimizing the wavenumber values. Although the IWC shows high accuracy in extracting complex wavenumbers, its disadvantages are that it employs a peak-searching algorithm which requires the setting of a threshold and that it is not capable of determining the number of modes. Especially for cases where higher-order Lamb modes are available, the determination of the complex wavenumbers becomes more sophisticated, and the IWC algorithm becomes computationally inefficient, in contrast to the MPDM or ESPRIT methods which become more convenient for such cases. To select the best algorithm between the two, the effect of noise in the data plays a crucial factor. As shown in the literature, the total least square ESPRIT (TLS-ESPRIT) gives much better results for imaginary wavenumbers compared to the direct MPDM when the noise level is above a certain threshold [33]. Nevertheless, for real wavenumber extraction, the error rates are very much similar for both methods in the presence of noise, while the MPDM is found to be much faster compared to ESPRIT. Therefore, in this study, MPDM is selected because of its superior speed and comparable accuracy to extract real wavenumbers. The flowchart of the MPDM algorithm is visualized in Figure 4.

Classically, measured signals x(r,t) are composed of a combination of several Lamb wave mode contributions and measurement noise. Even though the number of Lamb wave modes in a plate could in theory be infinite, only a couple of them can actually be observed within the selected frequency range. To estimate the effective number of modes, termed *K*, these signals need to be decomposed. Several steps are carried out. Firstly, signals are converted from the time domain to the frequency domain using a fast Fourier transform. Then, for each selected frequency ω, a Hankel matrix is constructed from the signals (x(r,ω)). This Hankel matrix is subsequently split into two different matrices $X_{1}\left(r_{1},\omega \right)$ and $X_{2}\left(r_{2},\omega \right)$ as follows:

(7) $$\begin{array}{cc} X_{1}\left(r_{1},\omega \right) & =\left[\begin{array}{cccc} x(r_{1},\omega ) & x(r_{2},\omega ) & \cdots & x(r_{H},\omega )\\ x(r_{2},\omega ) & x(r_{3},\omega ) & \cdots & x(r_{H+1},\omega )\\ \vdots & \vdots & \ddots & \vdots \\ x(r_{M-H},\omega ) & x(r_{M-H+1},\omega ) & \cdots & x(r_{M-1},\omega ) \end{array}\right] \end{array}$$

(8) $$\begin{array}{cc} X_{2}\left(r_{2},\omega \right) & =\left[\begin{array}{cccc} x(r_{2},\omega ) & x(r_{3},\omega ) & \cdots & x(r_{H+1},\omega )\\ x(r_{3},\omega ) & x(r_{4},\omega ) & \cdots & x(r_{H+2},\omega )\\ \vdots & \vdots & \ddots & \vdots \\ x(r_{M-H+1},\omega ) & x(r_{M-H+2},\omega ) & \cdots & x(r_{M},\omega ) \end{array}\right] \end{array}$$

where *M* denotes the number of measurement points in the space domain and *H* is the so-called pencil parameter the value of which is generally chosen between M/3 and M/2 [28]. In this study, it is considered fixed at M/2. The relationship between the $X_{1}$ and $X_{2}$ matrices is naturally linked to the wave propagation function and can be expressed as:

(9) $$X_{2}-X_{1}e^{-ik\Delta r}=0,k\in C$$

where Δr is the spatial distance between two subsequent measurement points. To find the wavenumber *k*, the roots of the equation need to be found. Therefore, $X_{1}$ is decomposed into primary complex exponentials via the singular value decomposition method:

(10) $$X_{1}=V_{L}\Sigma V_{R}^{*}$$

where the superscript * represents the Hermitian transpose of a matrix, and $V_{L}$ and $V_{R}$ are the left and right singular vectors, respectively. Σ is a diagonal matrix ($diag(\sigma_{1},\sigma_{2},\ldots$)) which contains the non-negative singular values in descending order. The highest set of singular values generally represents the actually present wave modes, while the lower singular values correspond to noise features. At this point, in order to separate noise from wave modes, a threshold value needs to be defined. As there is not a standardized way to define this value, it generally needs to be selected for each measurement individually. In this study, however, the threshold level is fixed and defined at a level of −50 dB compared to the first eigenvalue for both the numerical COMSOL simulations and the experiments. After setting the threshold value, the number of effective wave modes (K) is obtained, and the remaining singular values (min(H,M−H)−K) are considered to represent noise features. In the remaining analysis, only the first K columns of the left and right singular vectors are taken into account, expressed as $\tilde{V_{L}}$ and $\tilde{V_{R}}$. With this interpretation, Equation (9) can be rewritten and simplified as:

(11) $$\begin{array}{c} 0={\tilde{V_{L}}}^{*}X_{2}\tilde{V_{R}}-e^{-ik\Delta r}\tilde{\Sigma } \end{array}$$

where $\tilde{\Sigma }$ is again a diagonal matrix which now obviously only contains the first *K* largest wave modes. The complex valued eigenvalues, λ, of the reduced (K×K) matrix *Z*, which is defined as

(12) $$Z={\tilde{\Sigma }}^{-1}{\tilde{V_{L}}}^{*}X_{2}\tilde{V_{R}}$$

therefore correspond to the complex exponential term $e^{-ik\Delta r}$. Indeed, if $E_{\lambda }$ is an eigenvector for the eigenvalue λ of *Z*, then

(13) $$ZE_{\lambda }=\lambda E_{\lambda }\;or\;\left[{\tilde{\Sigma }}^{-1}{\tilde{V_{L}}}^{*}X_{2}\tilde{V_{R}}\right]E_{\lambda }=\lambda E$$

and thus $\lambda =e^{-ik\Delta r}$.

Finally, the real and imaginary parts of the pertinent wavenumber values can be calculated from these eigenvalues (λ) as follows:

(14) $$\left\{\begin{array}{c} k_{r}=\frac{-Im[ln(\lambda )]}{\Delta r}\\ k_{i}=\frac{Re[ln(\lambda )]}{\Delta r} \end{array}\right.$$

The calculated real wavenumber values for each frequency within the considered range are then stored and used as input in the inversion algorithm for reconstructing of the elastic stiffness tensor.

## 4. Results and Discussion

In the following subsections, various case studies are discussed with specific attention to the added value of including both the in- and out-of-plane velocity data in the inversion procedure. In each case, the median values and the median absolute deviation for the inverted stiffness tensor (after 20 PSO repetitions), and the deviation from the literature values if available, are reported for only in-plane motion, only out-of-plane motion, and full-field motion. The first two studies apply to the inversion of synthetic datasets generated for homogenized orthotropic materials with typical examples of a wooden plate (beech wood) and a C/E plate. In these cases, literature values for the elastic constants are used as input parameters for data generation, and as target elasticity values to compare with the inversion results. Furthermore, the effect of noise addition on the post-processed wavenumber data on the quality of the stiffness inversion is discussed. Subsequently, two experimental datasets acquired on a layered C/E plate and a woven G/F plate, were analyzed and inverted to determine their homogenized orthotropic elastic constants.

### 4.1. Numerical Case Study 1: Homogeneous Wooden Plate

**Figure 5:**
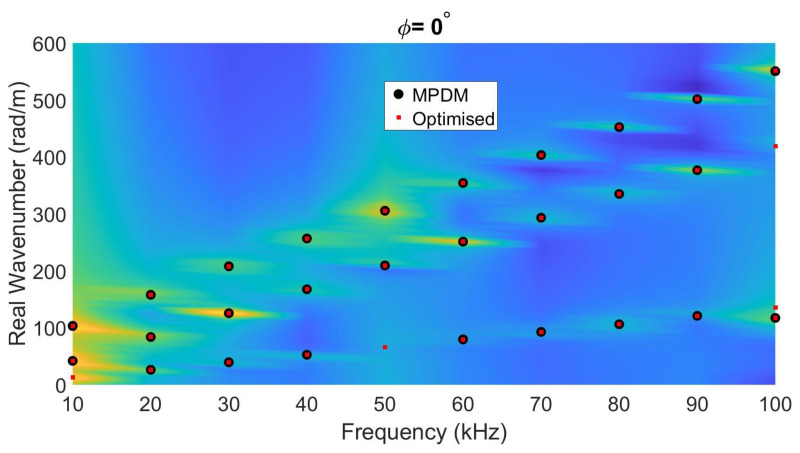
Wavenumber–frequency pairs calculated via the optimized stiffness values (using SAFE, red dots), compared to the MPDM-extracted wavenumber–frequency pairs (black open circles), extracted from FEM simulation on a 5 mm-thick wooden plate with orthotropic nature, and overlayed on the dispersion curves resulting from the simulated full-motion COMSOL data when ϕ=0.

In the first case study, a homogenized orthotropic wooden plate (beech wood) with a thickness of 5 mm and a density of 674 kg/m${}^{3}$ is considered. Details on the stiffness tensor can be found in Bucur and Rocaboy [34], and are listed in the column 2 of Table 1. The lamb wave propagation data were numerically simulated in the frequency range of 10–100 kHz, and analyzed using MPDM to provide the wavenumber spectrum on a 10 kHz frequency grid. The median and median absolute deviation of the inverted stiffness tensor components and the percentage deviation compared to the literature values for only in-plane motion, only out-of-plane motion, and full-field motion are listed in Table 1. The average error levels are 3.8%, 4.7%, and 3.6%, respectively. When using the full-field motion data, the overall accuracy increases and using in-plane data almost as good compared to full-field motion. The median absolute deviations in the inversion results are almost negligible in all cases. In this case, the lowest percentage errors are obtained for the shear stiffness parameters ($C_{44}$, $C_{55}$, and $C_{66}$), while the largest errors are observed for the out-of-plane stiffness parameters ($C_{13}$, $C_{23}$, and $C_{33}$). Even though the error percentages of these out-of-plane stiffness parameters are still quite high, their median absolute deviations are negligible. To better understand this, the dispersion curves corresponding to the full-field Lamb wave modeling for the in-plane direction ϕ=0 are depicted in Figure 5. The graph is obtained by taking the 1D Fourier transform of the data obtained from the COMSOL frequency domain analysis, i.e., transforming (x,f) data into (k,f) space. As the optimized results (red dots) perfectly match with the MPDM-based extracted wavenumber values (black open circles) and the calculated dispersion curves, this implies that the sensitivity of the out-of-plane stiffness parameters is extremely low within this *fd* regime (50–500 kHz·mm).

Within this *fd* regime, beech wood has only three wave modes, namely $A_{0}$, $S_{0}$, and $SH_{0}$, which is insufficient to identify the out-of-plane stiffness parameters with a sufficiently high accuracy. The sensitivity studies conducted for wave modes by Kudela et al. also shows that some stiffness parameters are not sensitive in the low frequency range [10]. To overcome this problem, the inversion process has been repeated for a frequency range between 10 and 200 kHz with 10 kHz grids. The inversion results for this higher *fd* range are shared in Table 2. The results show an average error rate of 0.12% for input information based on only in-plane motion, 0.16% for only out-of-plane motion, and 0.10% for full-field motion. The use of a wider frequency range thus obviously and spectacularly decreases both the error level. The median absolute deviations in both cases are extremely small, and it is not possible to compare the precision of the three velocity components on inversions. The improvement in error levels can be readily explained by the increase in the number of potential Lamb wave modes ($A_{0}$, $A_{1}$, $S_{0}$, $S_{1}$, and $SH_{0}$-$SH_{2}$) and the extra information about their cut-off frequencies. The sensitivity of the out-of-plane stiffness parameters is indeed extremely highly linked to the cut-off frequencies and to the presence of higher modes. While the extension of the *fd* range is quite simple to obtain in numerical simulations, it is not always the case in actual experiments. Indeed, it is important to note that for experiments in the *fd* range above 1500 kHz·mm, high levels of noise at higher sampling rates can be introduced in the laser Doppler vibrometer measurements on top of the frequency limitations of the piezoelectric actuators. Therefore, the upper frequency × thickness value attainable in the experimental measurements is limited in this study (particularly for the examples discussed in Section 4.4 and Section 4.5).

### 4.2. Numerical Case Study 2: Homogeneous C/E Plate

In the second case study, the numerically calculated Lamb wave dataset relates to a homogenized orthotropic carbon epoxy plate with a thickness of 5 mm and a density of 1571 kg/m${}^{3}$. Details on the stiffness tensor can be found in Deschamps and Hosten [35] and are listed in column 2 of Table 3. The excitation again consists of a broadband sine sweep signal with frequencies between 10 kHz and 100 kHz, and the recorded signals were analyzed using MPDM on a 10 kHz grid. The results of the three-way inversion procedures are given in Table 3. The average error levels for only in-plane motion, only out-of-plane motion, and full-field motion are 4.9%, 4.5%, and 4.5%, respectively. The results show that, even though the global error percentages are similar, the use of the full-field motion information again has the smallest error rates. Additionally, even though the selected *fd* regime again only has information about the first three fundamental modes ($A_{0}$, $S_{0}$, and $SH_{0}$), similarly to the first case study, the percentage error rate of the stiffness parameters in case 2 is much higher. Because the information that can be obtained from the same *fd* regime is smaller compared to that in the first case study due to the relatively high stiffness parameters of case 2. Thus, the median absolute deviation in the results are almost higher even for the full-field motion-based inversion. Therefore, the inversion process is again repeated for a wider frequency range between 15 kHz and 300 kHz with a 15 kHz MPDM analysis grid. The results are shared in Table 4 and reveal a significant amelioration with an average error rate of 0.09% for only in-plane motion, 0.15% for only out-of-plane motion, and 0.09% for full-field motion which is due to the extra information on higher order Lamb waves and their cut-off frequencies. Additionally, the median absolute deviation values become zero.

The above discussed numerical case studies use the calculated velocity signals without considering the impact of signal noise. To explore the robustness of the inversion routine, an additional study was conducted to assess the effect of noise on the inversion process.

### 4.3. Effect of Noise

In real life, actual recorded signals always contain a finite amount of noise due to environmental fluctuations (e.g., temperature and humidity), measurement conditions (e.g., surface roughness and curvature, dust), and hardware limitations. To investigate the effect of noise on the inversion process, random noise values are added to the extracted wavenumber values on each axis for the first case study (wooden plate).

**Figure 6:**
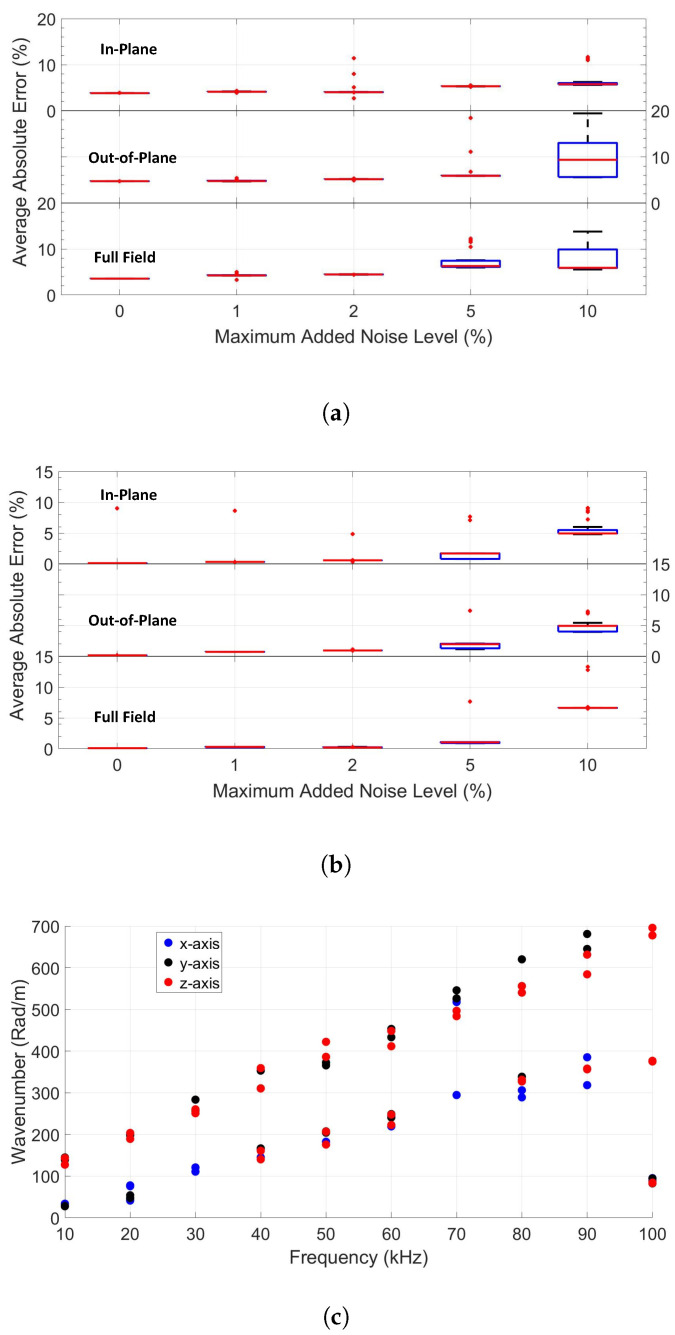
Illustrations of the effect of noise on the quality of the inversion results for beech wood (cfr case study 1): Measures of accuracy (average error percentage) and precision (median deviation) for different noise levels considering the *fd* range of 50–500 kHz·mm (**a**) and the *fd* range 50–1000 kHz·mm (**b**). Visualization of the extracted wavenumber–frequency pairs of beech wood for different velocity axes when ϕ=60 in the presence of 10% noise (**c**).

The quality of the inversion results in terms of the average absolute error percentage and the median deviation values of the stiffness components for different maximum noise ratios can be seen in Figure 6a (considering the *fd* range of 50–500 kHz·mm) and Figure 6b (for the *fd* range of 50–1000 kHz·mm). In these figures, the median absolute error percentages are indicated with the central mark (red horizontal bar), and the 25 and 75 range percentage deviations of the data are represented by the bottom and top edges of the blue colored box, respectively. Outlier points are marked with a red + symbol. The results are displayed for all of the three-way inversion procedures. One can readily deduce from the figures that the median absolute deviation as well as the average error values increase with increasing noise, especially for the low frequency inversion (50–500 kHz·mm, Figure 6a). The median absolute deviations (represented by the box sizes) are lower for inversions considering the information of only the in-plane motion compared to others, but the number of outlier results are also higher. On the other hand, the full field motion-based inversion gives higher accuracy (represented by lower error percentages) and less outlier results. The higher accuracy of the full field-based inversion can be explained as an averaging effect. As illustrated in Figure 6c, the extracted wavenumber–frequency pairs deduced from each measurement axes by MPDM can be slightly different from one another. Moreover, due of the presence of noise, it is also possible to extract multiple (two) wavenumber values for the same mode in the same axes dataset. As a result, the application of the full field motion-based inversion helps to average out the contribution of different wavenumbers associated with the same mode and thereby increases the accuracy of the results.

**Figure 7:**
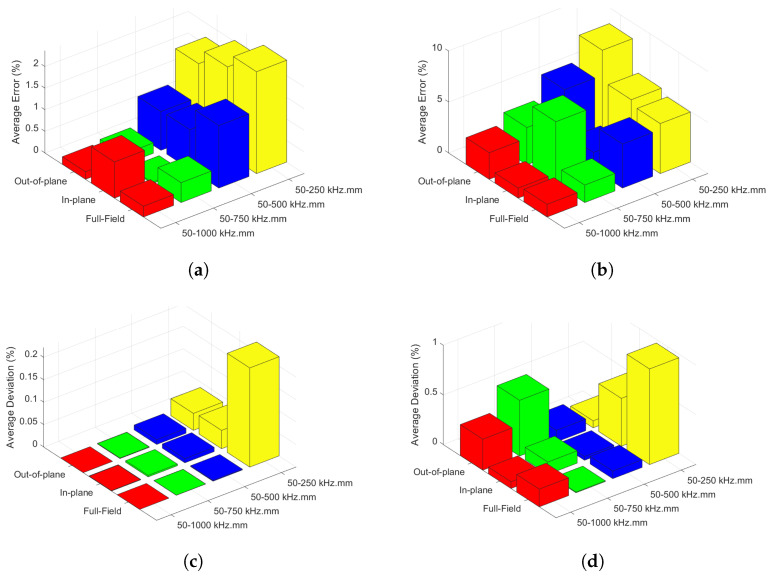
The median absolute percentage error of the elastic stiffness parameters (**a**) in the absence of noise, (**b**) in the presence of 5% noise, and the median absolute deviations in percentage (**c**) in the absence of noise, (**d**) in the presence of 5% noise, as a function of the velocity components taken into account in the inversion and the *fd* range.

To further validate the accuracy of the statement, an additional study was conducted on beech wood in the absence of noise and in the presence of 5% noise, and the accuracy (represented by average error percentages) as well as the median absolute deviations with respect to *fd* range and velocity components were examined (see Figure 7a–d). The results show that the accuracy and deviations are generally better when in-plane motion is used to identify elastic parameters in the presence of 5% noise. However, the effects of velocity components are rather limited on the accuracy of the results in the absence of noise, and the median absolute deviations are almost negligible for all *fd* range and velocity components.

This study thus clearly shows that, as expected, the accuracy and the precision of the inversion decreases when the noise level increases. Nonetheless, as long as the noise ratio is smaller than 10%, the error rates and the median absolute deviations are within an acceptable range, leading to the conclusion that the impact of noise is limited on the determination of the real wavenumber values. The effect of noise on the extraction of the imaginary wavenumbers needs to be further investigated in view of complete viscoelastic material characterization.

### 4.4. Experimental Case Study 1: Homogenized C/E Plate

In the first experimental case study, Lamb wave propagation data were collected on a 330 × 330 mm${}^{2}$ quasi isotropic C/E with a thickness of 2 mm and a density of 1572 kg/m${}^{3}$ in the frequency range of 5–300 kHz. The stacking sequence corresponds to [45/0/–45/90]${}_{s}$. To start up the inversion with reasonable estimates of the stiffness components, the stiffness parameters of a single lamina were taken from the literature [7], and the homogenized stiffness matrix was calculated by accounting for the layer orientation and layer thickness values [36]. The ultimately inverted stiffness parameters using the three-way inversion are summarized in Table 5. Even though we cannot really assess the accuracy of the three inversions, overall, the precision of the inverted constants is best in the case of full-field motion-based inversion (smallest median deviation values). Additionally, the literature values are also very similar to the full-field motion-based inversion results. A relatively strong variation in the reconstructed values for the out-of-plane, the in-plane and the full-field-based inversion results for certain stiffness parameters, especially for $C_{n3}$ (n=1…3) and $C_{66}$ is obtained for this quasi isotropic C/E plate. Indeed, as shown in the numerical case studies, in the absence of higher wave modes, the deviation between the out-of-plane, on the one hand, and the in-plane and full-field-based inversion results, on the other hand, can be relatively large. The reason behind these deviations can be explained by missing wave modes in certain propagation directions when only out-of-plane motion is used. Insufficient information about certain wave modes leads to large errors as well as high median absolute deviations. As it can be seen from Table 5, the in-plane and full-field motion based inversions are closely aligned with substantial differences compared to the out-of-plane motion-based inversions, which is similar to the results shared in Figure 6a,b. Moreover, as the acquired data were quite noisy in this experiment, this may lead to some spurious points which might affect the accuracy of the inversion. This problem can be eliminated by simply adopting an advanced noise filter or higher noise thresholding in MPDM.

**Figure 8:**
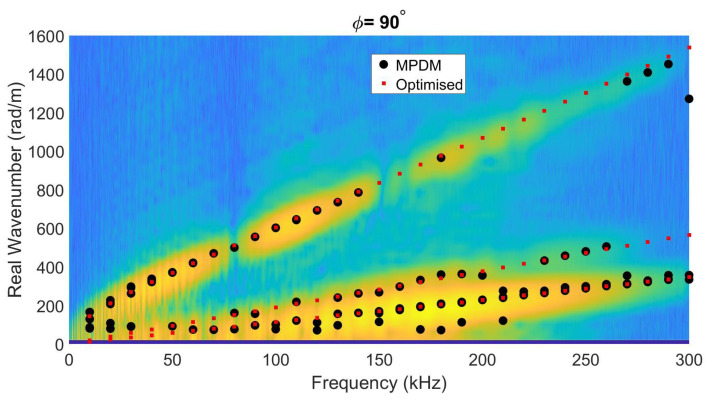
Wavenumber–frequency pairs calculated via the optimized (homogenized) stiffness values (using SAFE, red dots) compared to the MPDM wavenumber–frequency pairs (black open circles) extracted from an experiment on a 2 mm-thick quasi isotropic C/E plate, and overlayed on the dispersion curves resulting from the experimentally obtained full-motion data when ϕ=90.

In addition to the variation between the inverted values from the three inversions, the results shown in Table 5 also reveal a substantial difference between the homogenized estimates and the reconstructed values. This deviation is partly due to the difference between the samples and materials used in our study and in other cases in the literature, and partly to the homogenization calculation of the parameters which only provides a rough estimate for a layered medium when starting from the unidirectional ply parameters. This is conform with recent literature reports [11,37] where the inverted stiffness parameters ($C_{12}$, $C_{13}$ and $C_{23}$) were found to be almost 2.5 times higher than homogenized stiffness values, and also in line with the information coming from different manufacturers stating that the deviation in material properties (which were used as the basis for the homogenization calculation) might amount up to 10%. In order to validate the accuracy of the inversion results, the SAFE-based calculated wavenumber–frequency pairs corresponding to the optimized stiffness parameters of full-field motion inversion (red dots) are plotted in Figure 8, together with the MPDM-based extracted values from the experimental recordings (black open circles) and the experimentally obtained dispersion curves obtained through the 2D FFT of the star-like gridded laser Doppler vibrometer data, transforming (x,t;θ) data into (k,f;θ). Within the selected *fd* range, only three different wave modes can be observed and used for the inversion process, similarly to both numerical case studies discussed earlier.

### 4.5. Experimental Case Study 2: Homogenized G/F Woven Plate

**Figure 9:**
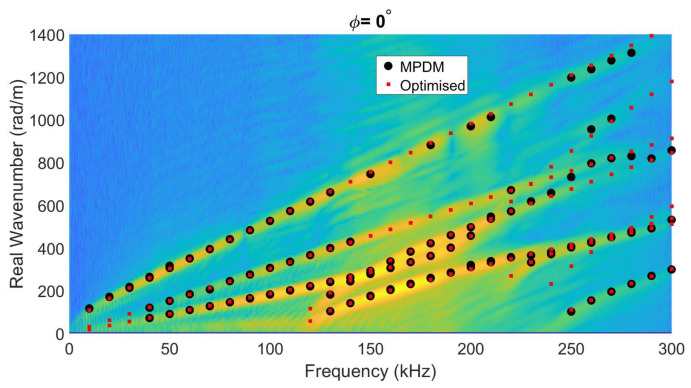
Wavenumber–frequency pairs calculated via the optimized (homogenized) stiffness values (using SAFE, red dots), compared to the MPDM wavenumber–frequency pairs (black open circles) extracted from an experiment on a 6 mm-thick unidirectional G/F woven plate, and overlayed on the dispersion curves resulting from the experimentally obtained full motion data when ϕ=0.

The fourth and final case study was conducted on a G/F woven plate (with a stacking sequence [#(45/–45)/#(0/90)]${}_{3s}$) with 6 mm thickness and 1730 kg/m${}^{3}$ density. Again, the inversion procedure started with C-tensor estimates based on the stiffness parameters of a single lamina taken from the literature [38], and the homogenized stiffness matrix calculated by using the proper stacking orientation and layer thickness values [36]. It should be noted that homogenization values may be incorrect because homogenization starts from unidirectional ply stiffness parameters. Moreover, the optimization procedure restarts the iteration if the components are found to be equal or close to the inversion boundaries, which explains the high difference between the theoretically calculated ’homogenized’ stiffness parameters and inverted ones. The final characterized stiffness parameters inverted based on the experimental measurements are listed in Table 6 for the three options. In contrast to the previous numerical test cases, the experimental data on this G/F woven plate comprise ten Lamb wave modes due to its larger thickness and the extended frequency range. Again, the application of the full-field motion information of Lamb waves for the (homogenized) C-tensor characterization significantly reduces the median absolute deviation of the inverted stiffness parameters. Additionally, literature values are similar to inverted stiffness values, and potential differences occur due to deviation angles between the material coordinate system and measurement coordinate system. To check the validity of the inversion results, the experimental dispersion curves (obtained through 2D FFT of the star-like gridded laser Doppler vibrometer data) and the MPDM extracted wavenumbers are again compared with the SAFE-based calculated wavenumber–frequency pairs corresponding to the optimized stiffness parameters of full-field motion inversion (red dots) in Figure 9. The figure qualitatively reveals a good match, especially for the higher-order Lamb modes.

## 5. Conclusions

In the present study, the effect of adding information to the in-plane polarized velocity components of a wavefield on top of the traditionally used out-of-plane component to the inversion of orthotropic elastic stiffness parameters is investigated. The inversion procedure uses the semi-analytical finite element (SAFE) method as a forward model to estimate the characteristic Lamb wavenumber–frequency pairs in a pre-set frequency–thickness (*fd*) range. Particle swarm optimization (PSO) was chosen for the inversion because of its speed and accuracy. The expected stiffness parameters are calculated by minimizing the absolute percentage error between the forward calculated (SAFE) wavenumbers and the wavenumbers deduced from actual or numerical wave propagation datasets using the matrix pencil decomposition method (MPDM). To obtain statistics on the results, the inversion procedure is repeated 20 times taking into account the heuristic nature of the optimization algorithm. The procedure is validated by using a series of COMSOL simulations and actual experiments on different materials. The numerical and experimental results show that it is possible to identify all nine stiffness parameters with an average error of less than 5%. Additionally, the study revealed that the identification of the out-of-plane stiffness ($C_{13}$, $C_{23}$, and $C_{33}$) parameters by only using $A_{0}$, $S_{0}$ and $SH_{0}$ modes might lead to high error rates (up to 13%). On the contrary, the shear stiffness values ($C_{44}$, $C_{55}$, and $C_{66}$) can be accurately and precisely identified (less than 0.3%, and small deviations). The identification problem for the out-of-plane stiffness components can simply be solved by extending the dataset to higher frequency–thickness ranges. Nevertheless, in case where only low-frequency thickness values can be examined, the additional information contained in the in-plane polarization does improve the accuracy while simultaneously reducing the median absolute deviation on the inverted stiffness parameters. It can be concluded that the knowledge of the in-plane velocity components is helpful in accurately determining the true stiffness values, and one can increase the accuracy and precision by either using the full motion or use large *fd* range. Additionally, the two experimental validation results proved that the full motion inversions are clearly better in terms of lower median deviations. Based on the case studies discussed in this paper, an inversion which takes into account the full-field motion can reduce the average error rates on the real valued C-tensor components up to 25% as well as lower the average median absolute deviations up to 92%.

## Figures and Tables

**Table 1:** PSO inversion results (in GPa) based on a numerically generated COMSOL dataset for a 5 mm-thick wooden plate with orthotropic nature, density 674 kg/m${}^{3}$, in the *fd* range of 50–500 kHz·mm. Partial (only in-plane or only out-of-plane) and full motion datasets were analyzed. The statistics (median and median absolute deviation) were performed using the output of 20 individual PSO simulations. The error rates are expressed in percentages (%).

C-Tensor Component	Actual Values	In-Plane Motion	Deviation (%)	Out-of-Plane Motion	Deviation (%)	Full-Field Motion	Deviation (%)
$C_{11}$	17.33	17.48 (±0.00)	0.90	17.17 (±0.00)	0.90	17.58 (±0.00)	1.45
$C_{12}$	3.03	3.10 (±0.00)	2.48	3.00 (±0.00)	0.51	3.11 (±0.00)	2.75
$C_{13}$	1.69	1.87 (±0.00)	10.47	1.74 (±0.00)	3.22	1.90 (±0.00)	12.22
$C_{22}$	3.26	3.29 (±0.00)	0.96	3.32 (±0.00)	1.64	3.28 (±0.00)	0.55
$C_{23}$	0.74	0.82 (±0.00)	9.87	0.87 (±0.00)	17.47	0.80 (±0.00)	7.17
$C_{33}$	1.64	1.79 (±0.00)	9.36	1.93 (±0.00)	17.47	1.76 (±0.00)	7.61
$C_{44}$	0.62	0.62 (±0.00)	0.03	0.62 (±0.00)	0.13	0.62 (±0.00)	0.03
$C_{55}$	1.09	1.09 (±0.00)	0.08	1.09 (±0.00)	0.07	1.09 (±0.00)	0.13
$C_{66}$	1.52	1.52 (±0.00)	0.07	1.54 (±0.00)	1.23	1.52 (±0.00)	0.07

**Table 2:** PSO inversion results (in GPa) based on a numerically generated COMSOL dataset for a 5 mm-thick wooden plate with orthotropic nature, with a density of 674 kg/m${}^{3}$, in the *fd* range of 50–1000 kHz·mm. Partial (only in-plane or only out-of-plane) and full-motion datasets were analyzed. The statistics (median and median absolute deviation) were performed using the output of 20 individual PSO simulations. The error rates are expressed in percentages (%).

C-Tensor Component	Actual Values	In-Plane Motion	Deviation (%)	Out-of-Plane Motion	Deviation (%)	Full-Field Motion	Deviation (%)
$C_{11}$	17.33	17.29 (±0.00)	0.20	17.29 (±0.00)	0.17	17.28 (±0.00)	0.25
$C_{12}$	3.03	3.02 (±0.00)	0.17	3.02 (±0.00)	0.08	3.02 (±0.00)	0.28
$C_{13}$	1.69	1.69 (±0.00)	0.10	1.70 (±0.00)	0.42	1.69 (±0.00)	0.06
$C_{22}$	3.26	3.26 (±0.00)	0.05	3.26 (±0.00)	0.04	3.26 (±0.00)	0.05
$C_{23}$	0.74	0.74 (±0.00)	0.44	0.74 (±0.00)	0.32	0.74 (±0.00)	0.13
$C_{33}$	1.64	1.64 (±0.00)	0.01	1.64 (±0.00)	0.14	1.64 (±0.00)	0.01
$C_{44}$	0.62	0.62 (±0.00)	0.05	0.62 (±0.00)	0.09	0.62 (±0.00)	0.05
$C_{55}$	1.09	1.09 (±0.00)	0.04	1.09 (±0.00)	0.01	1.09 (±0.00)	0.02
$C_{66}$	1.52	1.52 (±0.00)	0.01	1.52 (±0.00)	0.14	1.52 (±0.00)	0.00

**Table 3:** PSO inversion results (in GPa) based on a numerically generated COMSOL dataset for a 5 mm-thick C/E plate with orthotropic nature, a density of 1571 kg/m${}^{3}$, in the *fd* range of 50–500 kHz·mm. Partial (only in-plane or only out-of-plane) and full motion datasets were analyzed. The statistics (median and median absolute deviation) were performed using the output of 20 individual PSO simulations. The error rates were expressed in percentages (%).

C-Tensor Component	Actual Values	In-Plane Motion	Deviation (%)	Out-of-Plane Motion	Deviation (%)	Full-Field Motion	Deviation (%)
$C_{11}$	132	132.64 (±0.02)	0.48	132.31 (±0.02)	0.23	132.05 (±0.02)	0.04
$C_{12}$	6.9	7.43 (±0.01)	7.73	7.25 (±0.01)	5.12	7.21 (±0.02)	4.43
$C_{13}$	5.9	6.80 (±0.03)	15.21	7.06 (±0.03)	19.71	6.41 (±0.03)	8.69
$C_{22}$	12.3	12.69 (±0.01)	3.19	12.38 (±0.02)	0.63	12.71 (±0.02)	3.32
$C_{23}$	5.5	6.09 (±0.03)	10.78	5.83 (±0.04)	6.00	6.24 (±0.03)	13.49
$C_{33}$	12.1	12.81 (±0.05)	5.89	13.09 (±0.11)	8.21	13.37 (±0.07)	10.53
$C_{44}$	3.32	3.31 (±0.00)	0.34	3.32 (±0.00)	0.13	3.31 (±0.00)	0.26
$C_{55}$	6.21	6.20 (±0.00)	0.12	6.21 (±0.00)	0.05	6.20 (±0.00)	0.10
$C_{66}$	6.15	6.15 (±0.00)	0.04	6.18 (±0.00)	0.41	6.15 (±0.00)	0.04

**Table 4:** PSO inversion results (in GPa) based on a numerically generated COMSOL dataset for a 5 mm-thick C/E plate with orthotropic nature, density 1571 kg/m${}^{3}$, in the *fd* range of 75–1500 kHz·mm. Partial (only in-plane or only out-of-plane) and full motion datasets have been analyzed. The statistics (median and median absolute deviation) are performed using the output of 20 individual PSO simulations. The error rates are expressed in percentages (%).

C-Tensor Component	Actual Values	In-Plane Motion	Deviation (%)	Out-of-Plane Motion	Deviation (%)	Full-Field Motion	Deviation (%)
$C_{11}$	132	131.97 (±0.00)	0.02	131.73 (±0.00)	0.20	131.87 (±0.00)	0.10
$C_{12}$	6.9	6.91 (±0.00)	0.18	6.87 (±0.00)	0.47	6.90 (±0.00)	0.02
$C_{13}$	5.9	5.90 (±0.00)	0.01	5.89 (±0.00)	0.16	5.89 (±0.00)	0.10
$C_{22}$	12.3	12.28 (±0.00)	0.18	12.28 (±0.00)	0.13	12.28 (±0.00)	0.18
$C_{23}$	5.5	5.48 (±0.00)	0.36	5.48 (±0.00)	0.29	5.48 (±0.00)	0.35
$C_{33}$	12.1	12.10 (±0.00)	0.03	12.10 (±0.00)	0.03	12.10 (±0.00)	0.03
$C_{44}$	3.32	3.32 (±0.00)	0.04	3.32 (±0.00)	0.05	3.32 (±0.00)	0.05
$C_{55}$	6.21	6.21 (±0.00)	0.02	6.21 (±0.00)	0.04	6.21 (±0.00)	0.02
$C_{66}$	6.15	6.15 (±0.00)	0.00	6.15 (±0.00)	0.01	6.15 (±0.00)	0.00

**Table 5:** PSO inversion results (in GPa) based on a 3D infrared SLDV measurement dataset for a 2 mm-thick layered C/E plate with orthotropic nature, density of 1572 kg/m${}^{3}$, in the *fd* range of 10–600 kHz·mm. Partial (only in-plane or only out-of-plane) and full motion datasets were analyzed. The statistics (median and median absolute deviation) are performed using the output of 20 individual PSO simulations.

C-Tensor Component	Homogenized Estimated Values Based on Literature [7]	In-Plane Motion	Out-of-Plane Motion	Full-Field Motion
$C_{11}$	51.70	50.83 (±0.09)	45.46 (±0.14)	55.99 (±0.02)
$C_{12}$	18.40	24.28 (±0.06)	29.44 (±0.00)	26.18 (±0.01)
$C_{13}$	6.70	7.97 (±0.04)	4.05 (±0.03)	8.78 (±0.01)
$C_{22}$	51.70	60.63 (±0.08)	47.59 (±0.05)	62.64 (±0.01)
$C_{23}$	6.69	9.97 (±0.07)	4.03 (±0.02)	10.33 (±0.00)
$C_{33}$	13.73	8.91 (±0.04)	21.76 (±0.21)	8.48 (±0.00)
$C_{44}$	4.22	2.56 (±0.00)	2.75 (±0.00)	2.69 (±0.00)
$C_{55}$	4.22	2.54 (±0.00)	2.73 (±0.00)	2.63 (±0.00)
$C_{66}$	16.65	17.27 (±0.00)	9.99 (±0.00)	16.57 (±0.00)

**Table 6:** PSO inversion results (in GPa) based on a 3D infrared SLDV measurement dataset for a 6 mm-thick G/F woven plate with orthotropic nature, density 1730 kg/m${}^{3}$, in the *fd* range of 30–1800 kHz·mm. Partial (only in-plane or only out-of-plane) and full-motion datasets were analyzed. The statistics (median and median absolute deviation) are performed using the output of 20 individual PSO simulations.

C-Tensor Component	Homogenized Estimated Values Based on Literature [38]	In-Plane Motion	Out-of-Plane Motion	Full-Field Motion
$C_{11}$	24.84	25.19 (±1.78)	22.11 (±0.04)	22.87 (±0.00)
$C_{12}$	5.10	9.38 (±0.11)	9.51 (±0.08)	9.34 (±0.00)
$C_{13}$	5.41	6.25 (±0.53)	4.97 (±0.01)	5.22 (±0.00)
$C_{22}$	24.84	23.53 (±0.45)	23.58 (±0.04)	23.55 (±0.00)
$C_{23}$	5.41	5.62 (±0.83)	5.62 (±0.01)	5.62 (±0.00)
$C_{33}$	12.30	11.22 (±1.40)	11.18 (±0.01)	11.26 (±0.00)
$C_{44}$	3.28	2.80 (±0.01)	3.12 (±0.01)	2.81 (±0.00)
$C_{55}$	3.28	2.75 (±0.07)	2.84 (±0.01)	2.78 (±0.00)
$C_{66}$	3.04	6.84 (±0.00)	6.77 (±0.06)	6.84 (±0.00)

## Data Availability

Not applicable.
